# Periprosthetic Fungal Infections in Severe Endoprosthetic Infections of the Hip and Knee Joint—A Retrospective Analysis of a Certified Arthroplasty Centre of Excellence

**DOI:** 10.3390/jof7060404

**Published:** 2021-05-21

**Authors:** Andreas Enz, Silke C. Mueller, Philipp Warnke, Martin Ellenrieder, Wolfram Mittelmeier, Annett Klinder

**Affiliations:** 1 Orthopaedic Clinic and Policlinic, University Medicine Rostock, 18057 Rostock, Germany; martin.ellenrieder@med.uni-rostock.de (M.E.); mittelmeier.w@googlemail.com (W.M.); annett.klinder@med.uni-rostock.de (A.K.); 2 Institute of Pharmacology and Toxicology, University Medicine Rostock, 18057 Rostock, Germany; silke.mueller@med.uni-rostock.de; 3 Institute for Medical Microbiology, Virology and Hygiene, University Medicine Rostock, 18057 Rostock, Germany; philipp.warnke@med.uni-rostock.de

**Keywords:** candidiasis, infection, disarticulation, periprosthetic joint infection, antimycotic, caspofungin

## Abstract

The treatment of periprosthetic joint infections (PJI), and especially of re-infections, poses a highly complex problem in orthopaedic surgery. While fungal infections are rare, they present a special challenge. The therapy is often protracted and based on limited evidence. A total of 510 hip and knee revision surgeries were analysed for the occurrence of bacterial and fungal PJI. In patients with PJI, the duration of the hospital stay and the incidence of disarticulation of the infected joint were recorded. Out of the analysed revision arthroplasties, 43.5% were due to PJI. Monomicrobial infection occurred in 55.2%, dual microbial infection in 21.4%, and polymicrobial (≥3 different bacterial or fungal species) infection in 17.2% of the cases. Overall, *Candida species* were detected in 12.4% cases. *Candida albicans* was the main fungal pathogen. In 6.9% of cases, disarticulation of the joint was the only option to control PJI. The detection of polymicrobial infection more than doubled in follow-up revisions and there was a strong association between detection of *Candida* infection and disarticulation (OR 9.39). The majority of fungal infections were mixed infections of bacteria and *Candida albicans*. The choice of a biofilm penetrating antimycotic, e.g., caspofungin, together with a sufficient standard procedure for detection and surgical treatment can help to control the infection situation. Fungal infection often proves to be more difficult to treat than anticipated and is more frequent than expected.

## 1. Introduction

The treatment of bacterial infections of endoprostheses is a major challenge for physicians and patients [1]. The patient’s risk of re-infection and mortality increases with successive revision surgeries [2,3]. In some clinical cases, joint disarticulation is the only possible option for controlling the infection and for saving the patient’s life [4,5]. While infection control in bacterial infections is quite successful [1,6], treatment of mycoses represent a significantly higher challenge to the clinician and is associated with a very high long-term effort [7]. *Candida*, which is the fourth most frequent pathogen involved in nosocomial bloodstream infections in the USA [8] and which is associated with high mortality rates, was reported to most commonly cause nosocomial infections in general surgery [9,10]. The majority of fungal periprosthetic joint infections (PJI), which are considered risk factors for failure of revision surgery [11], were also caused by *Candida species (Candida)* spp. [12]. However, due to the rareness of these infections, there are only few publications that focus on fungi in PJI treatment. Recommendations for treatment procedures are insufficient [13,14]. The updated German guidelines on the treatment of fungal infections confirm the limited availability of data for candidiasis and periprosthetic joint infections while recommending a antifungal therapy with fluconazole and echinocandins [15]. The aim of the current study was to evaluate the incidence of yeast infections in PJI; in particular, the impact of candidiasis on the successful therapy in PJI was analysed.

## 2. Materials and Methods

### 2.1. Patients

**Figure 1:**
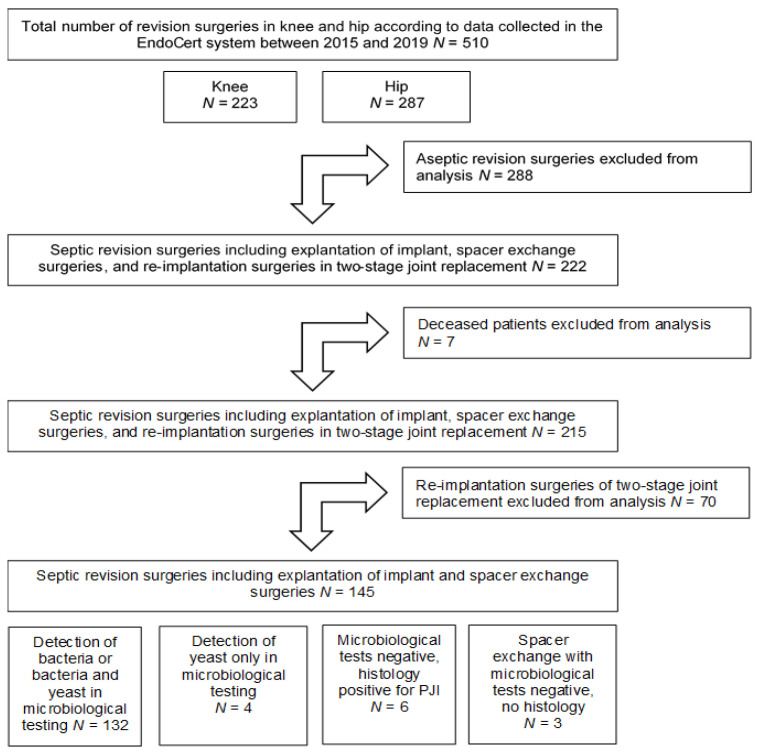
Scheme displaying the screening as well as the inclusion and exclusion of surgical procedures for the analysis regarding the incidence of fungal infections in PJI.

A retrospective analysis of patients who had undergone revision arthroplasty at the Orthopaedic Clinic and Policlinic of the University Rostock Medical Centre in the period from 19 January 2015 until 5 June 2019 was performed to determine the incidence of yeast infections in periprosthetic joint infections and to investigate the link between candidiasis in PJI and disarticulation. Of the total of 510 revision surgeries in this period, 288 surgeries in 253 patients were performed due to aseptic causes, while 222 surgeries in 124 patients were associated with PJI. Seven deceased patients were excluded from these 222 surgeries, while 70 re-implantation surgeries, which represented the second stage of two-stage revision arthroplasties, were excluded from the analyses of the bacterial and fungal spectrum, since the joints have to be pathogen-free before re-implantation can be carried out at the second stage (Figure 1). Thus, a total of 145 septic surgeries were analysed for the occurrence of bacterial and fungal pathogens in the joint. The total numbers of days per hospital stay, the incidence of disarticulation of the infected joint, and the ages of patients were also recorded for the 145 surgeries. The duration of the hospital stay was calculated as the period in which the patient stayed continuously at the University Rostock Medical Centre. Rehabilitation was not included in the hospital stay.

### 2.2. Treatment

Specimen sampling and surgical treatment were performed according to published guidelines for two-stage prosthetic replacement in PJI [1], including thorough debridement at all stages of the revision arthroplasty. Antibiotics were initially administered in a calculated regimen and were adapted accordingly after receiving the antibiogram of the causative pathogens. In the case of a detected fungal infection, the antifungal agent fluconazole or one of the echinocandin class was used according to the manufacturer’s instructions for dosing. Therapy was considered successful when wound conditions were dry and without signs of infection or swelling, when inflammatory markers (leukocytes, C-reactive protein, procalcitonin) approached normal values, and when the patient’s general condition stabilised to allow the discharge from our hospital.

### 2.3. Microbiological Testings

Microbiological analyses were performed at the Institute of Medical Microbiology, Virology, and Hygiene of the University Rostock Medical Centre. Microbiological diagnostics were carried out according to German microbiological standards [16,17] at the national accreditation organisation of the Federal Republic of Germany (DAkkS) DIN EN ISO 15,189- and DIN EN ISO/IEC 17,025-accredited microbiological laboratory.

### 2.4. Statistics

The results were collected using Microsoft Excel 2016 (Microsoft, Redmond, WA, USA). Statistical analyses were performed with IBM SPSS Statistics 27 (IBM Corp., New York, NY, USA), including descriptive statistics for continuous and categorical variables. Normal distribution was analysed by Shapiro–Wilk test. Cross-tables were generated to perform either Fisher’s exact tests or Chi square tests, as well as to calculate the odds ratios. The age distribution and number of days per hospital stay were compared using a Mann–Whitney U test. Here, *p*-values < 0.05 were considered as statistically significant. If not stated otherwise, all data are presented as means ± standard deviation (SD) (median; min–max).

## 3. Results

### 3.1. Patient Data

At 56.5% (288 surgeries in 253 patients), the majority of revision surgeries were carried out due to aseptic causes. Another 222 surgeries (43.5%) on 124 patients were associated with PJIs in the period from 2015 until 2019 (Figure 1). A total of 18 patients underwent revision surgeries for aseptic and septic causes.

In order to analyse the bacterial and fungal spectrum causing the infection, re-implantation surgeries, i.e., the second stage of two-stage revisions, were excluded from the analysis, as the joint is considered to be pathogen-free at this particular stage of the treatment of PJI. Additionally, the seven patients who died during their hospital stay were excluded to avoid bias regarding the duration of the hospital stay. None of the deceased seven patients had a fungal infection or underwent disarticulation. Causes of death were bacteremia (*Staphylococcus aureus*) with multi-organ failure, thromboembolic events, or complications due to the patients‘ pre-existing co-morbidities. Finally, 145 surgeries in a total of 110 patients were included for analysis. Out of the 145 surgeries, 80 were performed on the hip and 65 on the knee. The mean age of the patients was 70.2 ± 9.7 years (72.0, 43–88). 

### 3.2. Bacterial and Fungal Spectrum of the PJIs

The bacterial spectrum isolated from intraoperative samples is displayed in Table 1. Infections with one (mono-), two (bi-), or more than two (polybacterial) causative bacteria species were detected in 82 (56.6%), 28 (19.3%) and 22 (15.2%) surgeries, respectively, among the 132 surgeries with intraoperative detection of bacteria (see Figure 1). *Candida* spp. was detected intraoperatively in 18 of the 145 surgeries (12.4%). 

A total of 13 out of 110 (11.8%) patients were affected by candidiasis of the joint. In four intraoperative samples, *Candida species* were identified as the sole causative pathogen of the infection. Apart from *Candida* spp., no other fungal species were identified. While *Candida glabrata* and *Candida parapsilosis* were each detected only once, *Candida albicans* was detected 16 times during surgeries (Table 2), making it the fourth most identified pathogen in the 145 surgeries. However, the less common *Candida glabrata* and *Candida parapsilosis* were responsible for two of the four infections caused only by fungi—the other two were caused by *Candida albicans*, while the majority of infections involving *Candida albicans* (14×) were mixed infections of fungus and bacteria. *Candida albicans* was most commonly associated with coagulase negative staphylococci (*Staphylococcus epidermidis* 8×, *Staphylococcus capitis* 3×, *Staphylococcus hominis* 1×, and *Staphylococcus haemolyticus* 1×) and *Enterococcus faecalis* (6×), however there was no preference regarding mixed infections with one (6 surgeries, 42.9%), two (3 surgeries, 21.4%), or more than two bacteria species (5 surgeries, 35.7%). 

### 3.3. Association of Candidiasis of the Joint during PJI with Selected Patient Factors

Age distribution did not differ between patients with and without diagnosis of candidiasis of the joint, with mean ages of 70.2 ± 12.2 years (76.5, 43–83) and 70.2 ± 9.4 years (72.0, 43–88) at time of surgery, respectively, even when the median age was slightly higher in the candidiasis group. However, this increase was not significant (Mann–Whitney U test, *p* = 0.473). 

Joint candidiasis was mainly detected in patients with repeated revision surgeries. Candidiasis in the joint occurred in 83.3% during follow-up revisions, while the percentage of repeated revisions in patients without detection of *Candida species* was 42.5%. The risk to be afflicted by a *Candida* infection in the joint increased significantly (Fisher‘s exact t-test, *p* = 0.002), with odds ratios of 6.76 (95% CI: 1.86–24.52) when the revision surgery was a follow-up revision, i.e., the patient had undergone revision arthroplasty previously. A total of 50 out of the 110 patients (45.5%) had undergone repeated revisions and 69 of the 145 surgeries (47.6%) were follow-up revisions. 

The percentage of detection of polymicrobial infections more than doubled in follow-up revisions (27.3% compared to 10.0% in “primary” revisions; Pearson’s chi square test, *p* = 0.011). The percentage of infections involving three or more microbial species (polymicrobial) was also higher in the mixed joint infections of bacteria and *Candida species* compared to PJIs without yeast detection (44.4% vs. 14.4%; Pearson’s chi square test, *p* = 0.001). When defining infections with two or more microbial species as polymicrobial (Table 3), the increase in PJIs with yeast was even more pronounced (Fisher’s exact test, *p* = 0.001).

**Figure 2:**
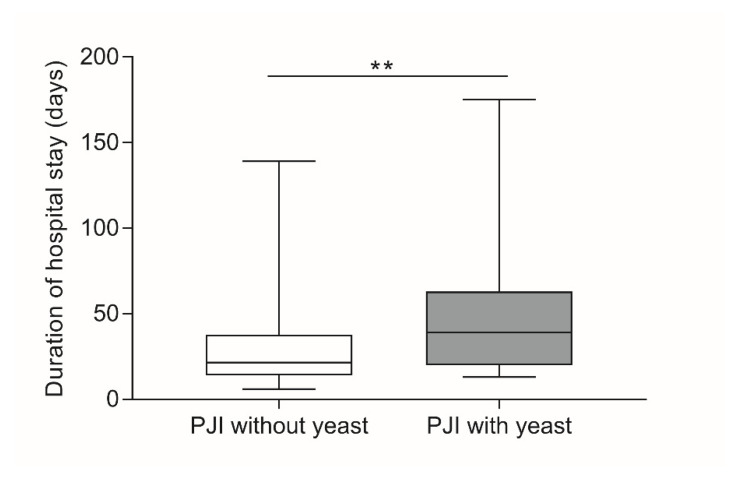
Hospital stays in days. Duration of the hospital stay was calculated as the period in which the patient stayed continuously at the University Rostock Medical Centre. Rehabilitation was not included in the hospital stay. The data are presented as boxplots with the minimum, 25th percentile, median, 75th percentile, and maximum. Significances between groups were calculated with the Mann–Whitney U test (** *p* < 0.01).

In the event of a *Candida* infection in the joint, the length of the hospital stay was significantly increased compared to PJIs without yeast detection (Mann–Whitney U test, *p* = 0.003), with average hospital stays of 54.8 ± 45.8 days (39.0, 13–175) and 27.9 ± 19.7 days (21.5, 6–139) (Figure 2), respectively. 

### 3.4. Fungal Infection and Disarticulation

Out of the 145 surgeries performed due to PJI, a total of 10 surgeries (6.9%) were disarticulations of the joint. All 10 disarticulations (100.0%) were carried out after repeated revisions and the percentage of follow-up revisions was, therefore, significantly higher than for the other revisions, where 43.7% (59 of 135 surgeries) were follow-up revisions (Fisher‘s exact t-test, *p* < 0.001). However, the odds ratio of the risk could not be calculated, as the value in the 2 × 2 table for disarticulation in “primary” revisions was zero.

**Figure 3:**
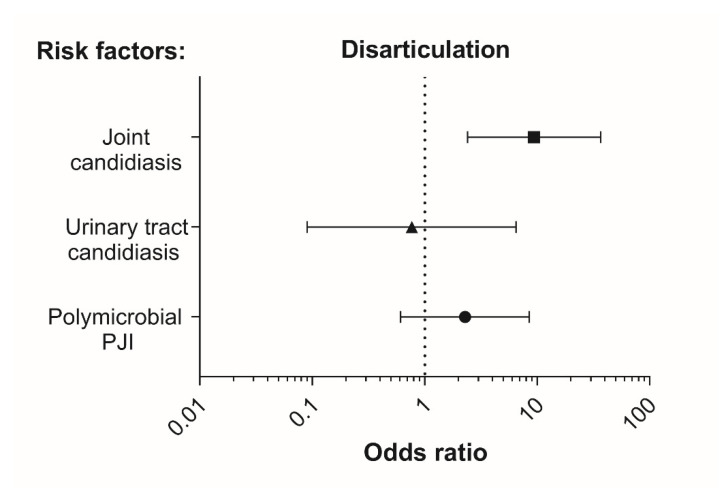
Association of disarticulation with microbial risk factors. Demonstration of the occurrence of disarticulation of the affected limb in relation to polymicrobial infection, candidiasis of the urinary tract, and candidiasis of the affected joint. Odds ratios are depicted as means ±95% confidence intervals.

In Figure 3, potential risk factors are depicted with their corresponding odds ratios. The detection of a *Candida* infection in the joint was significantly associated with disarticulation (Fisher‘s exact t-test, *p* = 0.003; OR 9.39 (95% CI: 2.40–36.75)). Out of the 18 PJIs with detection of *Candida species,* 5 cases (27.8%) were associated with disarticulation. In these 5 cases, PJI always presented as a mixed infection of *Candida albicans* with one (2×), two (2×), or four (1×) associated bacterial species (Table 2). In PJIs without yeast, the total number of disarticulations also comprised 5 cases; however, these only amounted to 3.9% (5 out of 127 cases). Hence, a clear link between disarticulation and candidiasis of the joint was observed.

To test the influence of the number of detected pathogens on the risk of disarticulation, PJIs were divided into infections caused by a single pathogen (monomicrobial) and infections caused by two or more pathogens, including *Candida species* (polymicrobial), in order to create a 2 × 2 table. Based on this allocation of PJIs into groups, a higher percentage of polymicrobial infections was recorded in association with disarticulation at 60.0% (6 out of 10) vs. 39.7% in the rest of the surgeries (50 out of 126). However, probably due to the small total number of disarticulations, this effect was not significant (Fisher‘s exact t-test, *p* = 0.317). The odds ratio was OR 2.28 (95% CI: 0.61–8.49). 

The age distribution of the patients in the disarticulation surgeries was also not different to the age of the patients in the rest of the surgeries (Mann–Whitney U test, *p* = 0.507).

### 3.5. Clinical Outcome after Fungal Infection

**Figure 4:**
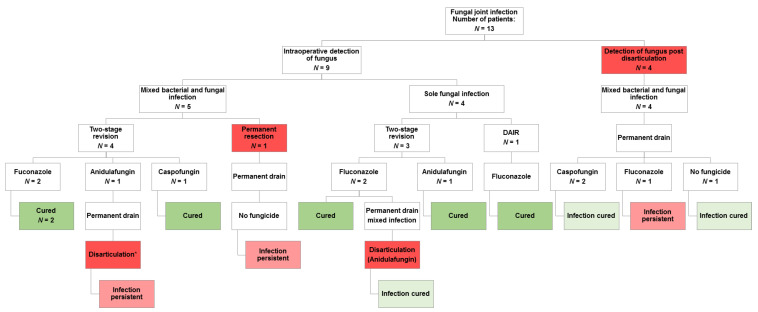
Detailed presentation of cases with periprosthetic infection and candidiasis.

Since a significant association between fungal infection and disarticulation was recorded, all available follow-up data for the 13 patients with fungal infections were analysed in order to elucidate whether there was a causative link between fungal infection and disarticulation. The clinical outcomes of the 13 patients with fungal infection are depicted in Figure 4. A total of 2 cases of the 9 intraoperatively detected fungal infections finally resulted in disarticulation (one occurred only in the follow-up, i.e., later than the initial analysis period until June 2019); at 22.2%, this is still a reasonably high percentage compared to the 3.9% in PJIs without fungal. However, the majority of fungal infections associated with disarticulation (*n* = 4) occurred after disarticulation. 

## 4. Discussion

The treatment of bacterial joint and bone infections in patients after multiple revision arthroplasties is very challenging, however the occurrence of opportunistic fungal infections is a further detrimental factor that complicates these infections [12]. The most commonly reported fungal pathogens are *C. albicans* and *C. glabrata* [13]. These two *Candida species* were also identified in the patients in this study.

### 4.1. Role and Occurrence of Fungal Infections in PJI

It is often reported that fungal infections are relatively rare, with rates being below 1%. However, in the most commonly cited publications by Azzam et al. and Hwang et al., the calculations of the incidence were based on the number of case reports in the literature or the number of primary total knee arthroplasties (TKA) in the centres, respectively [18,19]. In the relatively few publications that related the detection rate of fungi to PJIs in total, fungi were isolated in 0.9–2.0% of cases [12,20,21,22]. However, higher isolation rates were also reported, for example by Bori et al., who isolated *Candida species* in 2 out of 38 revision arthroplasties (5.3%) [23]. Assuming an incidence rate of 2% PJI after primary TKA [24], approximately 512 patients of the 25,585 primary TKAs in the study by Hwang et al. [19] would have developed a PJI. When then calculating the incidence of fungal PJIs based on the assumed number of PJIs, the 30 reported fungal PJIs would also amount to a percentage of 5.9% (30/512). While this is still lower than the values in the current study, where *Candida species* were isolated in approximately 12% of cases, the data here are in accordance with one of the previous studies by this centre, in which *Candida species* were isolated in 3 of 27 intraoperative samples of confirmed PJI [25]. The higher detection rates in this centre might be due to various reasons. Since this orthopaedic centre offers an outpatient clinic specifically for septic implant loosening and employs specialist surgeons for PJI, most of the difficult cases, often after several unsuccessful revision arthroplasties or treatment attempts, from the vicinity of up to 180 km around our hospital are referred to this unit. This may account for the relatively high occurrence of fungal pathogens in our patients. Additionally, the data collected here were not part of a population-based study, rather they only show the occurrence of candidiasis in PJI for our Certified Centre of Excellence for Joint Replacement. Thus, it is difficult to generalise our results. However, it may also be possible that the microbial spectrum in PJIs has been changing in recent years. Wang et al. showed that not only did the incidence of PJI decrease over a 13-year period (2002–2014) but the microbial spectrum changed too; amongst other factors, the isolation of *Candida species* changed, which increased from 0 via 2.5% to 2.8% in the consecutive time periods analysed during their study [26]. Thus, while PJI overall occurs less often, difficult-to-treat infections might be on the rise. In the past, the isolation of *Candida* spp. was regularly judged to be a contamination. For example, Brown et al. excluded 18 infections with detection of *Candida* as contaminations from their analysis [12]. However, in 2013, in accordance with Dutronc et al., Kuipers et al. concluded that a cultured fungal species should always be considered a pathogen [27,28]. They reported that in 21% of the patients, the fungal culture result was—incorrectly—considered to be a contamination. This is even more important in light of the fact that culture-based detection methods have sensitivities as low as 38% [29,30,31]. 

### 4.2. Biofilm Matrix Formation and Bacterial Symbiosis

Clinically, it might not be crucial whether *Candida* is the causing pathogen or an opportunistic pathogen after long antibiotic treatment. As soon as fungal species are detected, the situation in PJI becomes “difficult-to-treat”—even more so when there is a bacterial co-infection, as *Candida species* and selected bacteria species establish a mutually beneficial mixed biofilm that protects them from antimicrobial treatment. The bacterial species detected in our patients have all been described to form mutually beneficial mixed biofilms with *Candida* [32,33,34,35,36,37,38]. Staphylococci can affect the activity of antifungal drugs, while staphylococcal proteinase enhanced the adhesion ability of *C. albicans* [33], thus favouring the survival of the yeast. On the other hand, the presence of *Candida species* increased the growth of anaerobic bacteria by generating a hypoxic microenvironment [36], stimulated biofilm formation in charge-homogeneous *Enterococcus faecalis* strains, which are normally unable to form biofilm on their own [32], and led to enhanced tolerance of *Staphylococcus aureus* towards vancomycin [37]. Kong et al. speculated that the resistance to vancomycin was due to a barrier function of polysaccharides secreted by *Candida*, in particular β-1,3-glucans, which actually coat the bacteria and physically prevent an interaction between bacterial cell and antibiotic [37]. A similar mechanism was described for *Escherichia coli–Candida albicans* biofilms and ofloxacin [39]. This highlights the importance of establishing comprehensive treatment options for bacterial and fungal co-infections. 

### 4.3. Antifungal Therapy and Choice of Medication

The 2016 IDSA guidelines advise to use either fluconazole, an echinocandin, or liposomal amphotericin B in osteoarticular infections caused by *Candida species* [40], while the German Association of the Scientific Medical Societies recently recommended echinocandins in the treatment of PJI [15]. While a high fluconazole resistance was reported for *Candida albicans* biofilms, treatment with caspofungin was shown to decrease the ability of *Candida* *spp.* to form biofilms and to penetrate existing biofilms [41,42,43]. The biofilm penetrating effect is of special importance for the successful treatment of mixed bacterial and fungal infections. Since the majority of candidiasis in this study presented as long-lasting mixed infections of *Candida albicans* and bacteria that form mutually beneficial mixed biofilms with *Candida species*, the biofilm-penetrating capability of caspofungin could be crucial for success in treating PJI (Table S1). The observation that in mixed PJI a turnaround in the progression of infection was only achieved after commencing treatment with caspofungin suggests that only then was the susceptibility to the antibiotics restored and could antibiotic therapy successfully eradicate the bacterial pathogens [44].

### 4.4. Surgical Treatment Algorithm and Limits of Therapy

**Figure 5:**
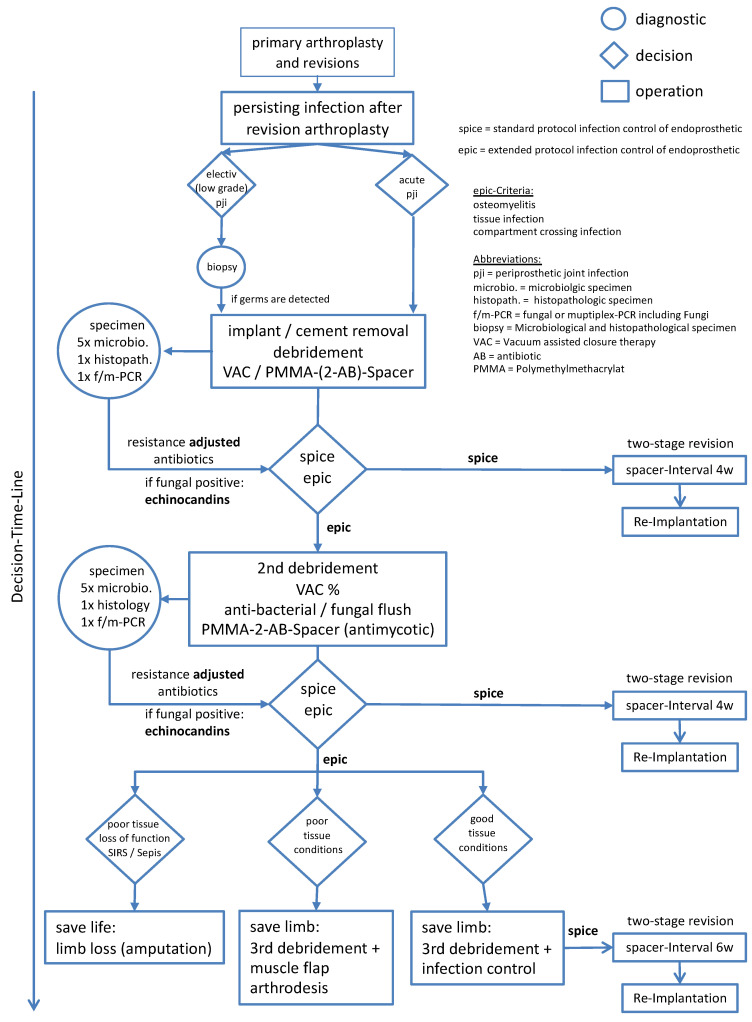
Therapy regimen. Scheme for the treatment of severe periprosthetic infections with the decision flow method for standardised therapy.

Treatment algorithms for bacterial infections of endoprostheses are well established [1], but there are only limited recommendations for the treatment of fungal joint infections [40]. Upon reflection on the data from the presented study, an algorithm was developed (Figure 5) for the identification and treatment of polymicrobial and fungal infections. A focused surgical approach is indispensable. In addition to surgical and pharmacological therapy, the diagnosis of a fungal infection presents a central problem. Targeted diagnostics are an important part of the treatment strategy, especially in cases of re-infection [45]. The proposed algorithm (Figure 5) must be further investigated and validated in prospective studies. The indication for amputation or disarticulation during this study was based on the extent and depth of the infection. The detection of candidiasis did not influence the indication for amputation. However, there might be a need to consider this in future decision making, both for and against amputation. The decision would depend on whether the fungus can be treated with a suitable antifungal agent. According to our study, the choice of antimycotic could play a central role in the preservation of the affected limb.

This study also highlights the detrimental outcome of a candidiasis of the joint, as the isolation of *Candida species* was linked with disarticulation. A follow-up for all 13 patients with *Candida* detection revealed that mixed infections of *Candida* and bacterial species often occurred as a result of disarticulation. However, with an incidence of 22.2%, they also represented a major cause of disarticulation. Previous studies also reported high rates of disarticulation due to treatment failure of *Candida* joint infections. In three stringent follow-up studies, disarticulation rates of 7.5% [28], 11.1% [46], and up to 17.2% [18] were reported. These are considerably higher than the disarticulation rate of 3.9% determined in our study in PJIs without yeast. This highlights the importance of treatment algorithms for fungal PJIs to avoid the loss of the infected limb. In this regard, prompt and reliable detection of an involvement of fungal microorganisms in PJI, which is often hampered by the low sensitivity of single detection methods [47,48], is essential for the success of the treatment.

## 5. Conclusions

The therapy of patients with bone or joint infection after endoprosthesis revision is very challenging. Mixed infections of bacteria and *Candida* considerably complicate the treatment and require a stringent, decisive procedure and appropriate experience. The influence of mixed infections on the activity of antifungal and especially on antibiotic drugs, i.e., the increased tolerance to those agents, has to be taken into consideration when treating the patient. The use of echinocandins together with a well-planned surgical standard treatment helped to control the infection situation and improved patient’ survival. We speculate that the successful treatment with caspofungin depends on its ability to restore the susceptibility of bacteria to antibiotics in these mixed infections. The detection method of fungi is crucial in identifying the presence of a fungal pathogen in a complex PJI. PCR methods might be a faster and more reliable option for detection of fungal pathogens. The proposed treatment algorithm should be investigated in further prospective studies.

## Figures and Tables

**Table 1:** Distribution of the isolated bacterial species.

Pathogen	Bacterial Spectrum	
	Total Number	Percentage (%)
*Staphylococcus epidermidis*	56	25,81
*Staphylococcus aureus* MSSA^+^	23	10.60
*Enterococcus faecalis*	22	10.14
*Cutibacterium acnes*	15	6.91
*Staphylococcus aureus* MRSA*	11	5.07
*Escherichia coli*	11	5.07
*Staphylococcus capitis*	9	4.15
*Staphylococcus hominis*	9	4.15
*Staphylococcus haemolyticu*	8	3.69
*Streptococcus agalactiae*	6	2.76
*Streptococcus dysgalactiae*	6	2.76
*Proteus mirabilis*	6	2.76
*Klebsiella pneumoniae*	4	1.84
*Pseudomonas aeruginosa*	4	1.84
*Staphylococcus lugdunensis*	3	1.38
*Enterobacter cloacae*	3	1.38
*Streptococcus mitis*	3	1.38
*Staphylococcus caprae*	2	0.92
*Enterococcus faecium*	2	0.92
Corynebacterium sp.	2	0.92
*Corynebacterium tuberulostearicum*	2	0.92
*Micrococcus luteus*	2	0.92
*Streptococcus anginosus*	1	0.46
*Corynebacterium striatum*	1	0.46
*Listeria monocytogenes*	1	0.46
*Cutibacterium avidum*	1	0.46
*Parvimonas micra*	1	0.46
*Finegoldia magna*	1	0.46
*Klebsiella oxytoca*	1	0.46
*Morganella morganii*	1	0.46

^+^ MSSA = methicillin-susceptible *S. aureus*; * MRSA = methicillin-resistant *S. aureus.*

**Table 2:** List of individual surgical procedures with intraoperative detection of fungal infections, including the bacterial spectrum for mixed infections.

*Candida species*	Joint	1. Bacterial Pathogen	2. Bacterial Pathogen	3. Bacterial Pathogen	4. Bacterial Pathogen	5. Bacterial Pathogen	Duration of Hospital Stay	Disarticulation	Age
*C. parapsilosis*	hip	none					19	no	81
*C. albicans*	hip	none					19	no	79
*C. glabrata*	knee	none					13	no	83
*C. albicans*	knee	none					46	no	67
*C. albicans*	hip	*S. epidermidis*					20	yes	76
*C. albicans*	hip	*S. epidermidis*					175 *	no	80
*C. albicans*	knee	*S. epidermidis*					175 *	no	80
*C. albicans*	hip	MRSA					28	no	66
*C. albicans*	hip	*E. coli*					74	no	59
*C. albicans*	hip	*K. pneumoniae*					59	yes	81
*C. albicans*	hip	*S. epidermidis*	*E. faecalis*				175 *	no	80
*C. albicans*	hip	*P. mirabilis*	*E. faecalis*				63	yes	52
*C. albicans*	knee	MRSA	*E. faecalis*				139	yes	54
*C. albicans*	hip	*S. epidermidis*	*S. capitis*	*E. coli*			175 *	no	80
*C. albicans*	hip	*S. hominis*	*S. capitis*	*P. aeruginosa*			33	no	43
*C. albicans*	hip	*S. epidermidis*	*E. faecalis*	MRSA	*S. agalactiae*		58	yes	77
*C. albicans*	hip	*S. epidermidis*	*E. faecalis*	*E. coli*	*S. haemolyticus*		39	no	63
*C. albicans*	hip	*S. epidermidis*	*E. faecalis*	*E. coli*	*S. aureus*	*M. morganii*	37	no	63

Duration of stay in days; age in years; MRSA = methicillin-resistant *S. aureus;* * = different surgical procedures with detection of *C. albicans* in the same patient as part of the same hospital stay with a total length of 175 days. For the statistical evaluation the duration of 175 days was only included once in the calculation of mean and median of the length of the hospital stay.

**Table 3:** Overview of patient and microbial factors in PJI without and with detection of a *Candida species*. ^§^ Mann–Whitney U test; ^#^ Fisher’s exact test.

Factor		PJI (Surgical Procedures)		*p*-Value
		w/o *Candida* *n* = 127	with *Candida* *n* = 18	
Joint	Hip	66 (52.0%)	14 (77.8%)	0.045 ^#^
	Knee	61 (48.0%)	4 (22.2%)	
Age(Years)	mean ± SD	70.2 ± 9.4	70.2 ± 12.2	0.473 ^§^
	median; Min–Max	72.0; 43–88	76.5; 43–83	
Duration of hospital stay (days)	mean ± SD	27.9 ± 19.7	54.8 ± 45.8	0.003 ^§^
	median; Min–Max	21.5; 6–139	39; 13–175	
Previous revisions	Yes	54 (42.5%)	15 (83.3%)	0.002 ^#^
Polymicrobial infection (≥2 microbial species including fungi)	Yes	42 (35.6%)	14 (77.8%)	0.001 ^#^
Disarticulation	Yes	5 (3.9%)	5 (27.8%)	0.003 ^#^

## Data Availability

Data are stored and available at the Orthopedic Clinic and Policlinic, University Medicine Rostock, Doberanerstraße 142, 18057 Rostock, Germany.

## Supplementary Materials

The following are available online at https://www.mdpi.com/article/10.3390/jof7060404/s1, Table S1. Relation of choice of antifungal agents to main outcome.
