# Supplementary material for: Periprosthetic Fungal Infections in Severe Endoprosthetic Infections of the Hip and Knee Joint—A Retrospective Analysis of a Certified Arthroplasty Centre of Excellence
Source: J Fungi (Basel). 2021 May 21;7(6):404. doi: 10.3390/jof7060404 (PMC8224054; doi:10.3390/jof7060404)
Supplement: Supplementary file 1 [file jof-07-00404-s001.zip › jof-1212680-supplementary.pdf]

Journal of Fungi

MDPI

Supplementary

Table S1. Relation of choice of antifungal agents to main outcome.

Outcome		Antifungal drug<br>Fluconazole	 Anidulafungin	 Caspofungin	p-Value<br>(chi-square)
**Infection**	**cured**	4 (66.7%)	2 (66.7%)	3 (100.0%)	0.361
	persistent	2 (33.3%)	1 (33.3%)	0 (0.0%)	
